# Choline transporter-like proteins 1 and 2 are newly identified plasma membrane and mitochondrial ethanolamine transporters

**DOI:** 10.1016/j.jbc.2021.100604

**Published:** 2021-03-28

**Authors:** Adrian Taylor, Sophie Grapentine, Jasmine Ichhpuniani, Marica Bakovic

**Affiliations:** Department of Human Health and Nutritional Sciences, University of Guelph, Guelph, Canada

**Keywords:** CTL1, CTL2, ethanolamine transport, phospholipids, membranes, Cho, choline, CK, choline kinase, CTL, choline transporter-like, DAG, diacylglycerol, EK, Etn kinase, Etn, ethanolamine, OCT, organics cation transporter, PC, phosphatidylcholine, PE, phosphatidylethanolamine, PS, phosphatidylserine, SLC44A, solute carrier 44A

## Abstract

The membrane phospholipids phosphatidylcholine and phosphatidylethanolamine (PE) are synthesized *de novo* by the CDP-choline and CDP-ethanolamine (Kennedy) pathway, in which the extracellular substrates choline and ethanolamine are transported into the cell, phosphorylated, and coupled with diacylglycerol to form the final phospholipid product. Although multiple transport systems have been established for choline, ethanolamine transport is poorly characterized and there is no single protein assigned a transport function for ethanolamine. The solute carriers 44A (SLC44A) known as choline transporter-like proteins-1 and -2 (CTL1 and CTL2) are choline transporter at the plasma membrane and mitochondria. We report a novel function of CTL1 and CTL2 in ethanolamine transport. Using the lack or the gain of gene function in combination with specific antibodies and transport inhibitors we established two distinct ethanolamine transport systems of a high affinity, mediated by CTL1, and of a low affinity, mediated by CTL2. Both transporters are Na^+^-independent ethanolamine/H^+^ antiporters. Primary human fibroblasts with separate frameshift mutations in the CTL1 gene (M1= *SLC44A1*^ΔAsp517^ and M2= *SLC44A1*^ΔSer126^) are devoid of CTL1 ethanolamine transport but maintain unaffected CTL2 transport. The lack of CTL1 in M2 cells reduced the ethanolamine transport, the flux through the CDP-ethanolamine Kennedy pathway, and PE synthesis. In contrast, overexpression of CTL1 in M2 cells improved ethanolamine transport and PE synthesis. These data firmly establish that CTL1 and CTL2 are the first identified ethanolamine transporters in whole cells and mitochondria, with intrinsic roles in *de novo* PE synthesis by the Kennedy pathway and intracellular redistribution of ethanolamine.

Phosphatidylcholine (PC) and phosphatidylethanolamine (PE) are major components of cellular membranes where they are involved with essential cellular processes (1, 2). PC and PE are synthesized *de novo* by CDP-Cho and CDP-Etn branches of the Kennedy pathway in which the extracellular substrates choline (Cho) and ethanolamine (Etn) are actively transported into the cell, phosphorylated, and coupled with diacylglycerols (DAGs) to form the final phospholipid product. Although multiple transport systems have been established for Cho, Etn transport is poorly characterized and there is no single gene/protein assigned a transport function for mammalian Etn.

Cho transport for membrane phospholipid synthesis is mediated by Cho transporter-like protein CTL1/SLC44A1 (3). CTL1 is the only well-characterized member of a broader family (CTL1-5/SLC44A1-5) (4, 5). CTL1/SLC44A1 is a Cho/H^+^ antiporter at the plasma membrane and mitochondria (4, 5). The role of plasma membrane CTL1 is assigned to Cho transport for PC synthesis, but the exact function of the mitochondrial CTL1 is still not clear. In the liver and kidney, mitochondrial CTL1 transports Cho for oxidation to betaine, the major methyl donor in the one-carbon cycle (6). In other tissues, however, the mitochondrial CTL1 probably maintains the intracellular pools of Cho and as a H^+^-antiporter modulates the electrochemical/proton gradient in the mitochondria (7, 8). CTL2/SLC44A2 is only indirectly implicated in PC synthesis and its exact function is not firmly established in either whole cells or mitochondria (4).

PE is the major inner membrane phospholipid with specific roles in mitochondrial fusion, autophagy, and apoptosis (9, 10, 11). PE is also a valuable source of other phospholipids. PC is produced by methylation of PE, whereas phosphatidylserine (PS) is produced by an exchange mechanism whereby the Etn moiety of PE is replaced with serine and free Etn is released. PC could also produce PS by a similar exchange mechanism, with free Cho being released. The metabolically released Cho and Etn need to be transported in and out of the cytosol and mitochondria or reincorporated into the Kennedy pathway (3, 4, 5, 6). That mammalian Etn and Cho transport may occur through a similar transport system was implicated from early kinetic studies in bovine endothelial cells, human retinoblastoma cells, and glial cells (12, 13, 14). Here, we demonstrate that CTL1/SLC44A1 and CTL2/SLC44A2 are authentic Etn transporters at the cell surface and mitochondria. We examined the kinetics of Etn transport in CTL1 and CTL2 depleted conditions and overexpressing cells. We characterized Etn transport in human skin fibroblasts that maintain CTL2 but lack CTL1 function due to inherited *CTL1/SLC44A1* frameshift mutations (M1= *SLC44A1* ^ΔAsp517^ and M2= *SLC44A1* ^ΔSer126^) (15). We employed pharmacological and antibody-induced inhibition to separate the contributions of the CTL1 and CTL2 to Etn transport and PE synthesis. This study is the first to demonstrate that the CTL1 and CTL2 are high- and low- to medium-affinity cellular and mitochondrial Etn transporters. To our knowledge, this is the first study to demonstrate that, as intrinsic Etn transporters, CTL1 and CTL2 regulate the supply of extracellular Etn for the CDP-Etn pathway, redistribute intracellular Etn, and balance the CDP-Cho and CDP-Etn arms of the Kennedy pathway.

## Results

### CTL1 and CTL2 inhibition reduces Etn and Cho transport

**Figure 1:**
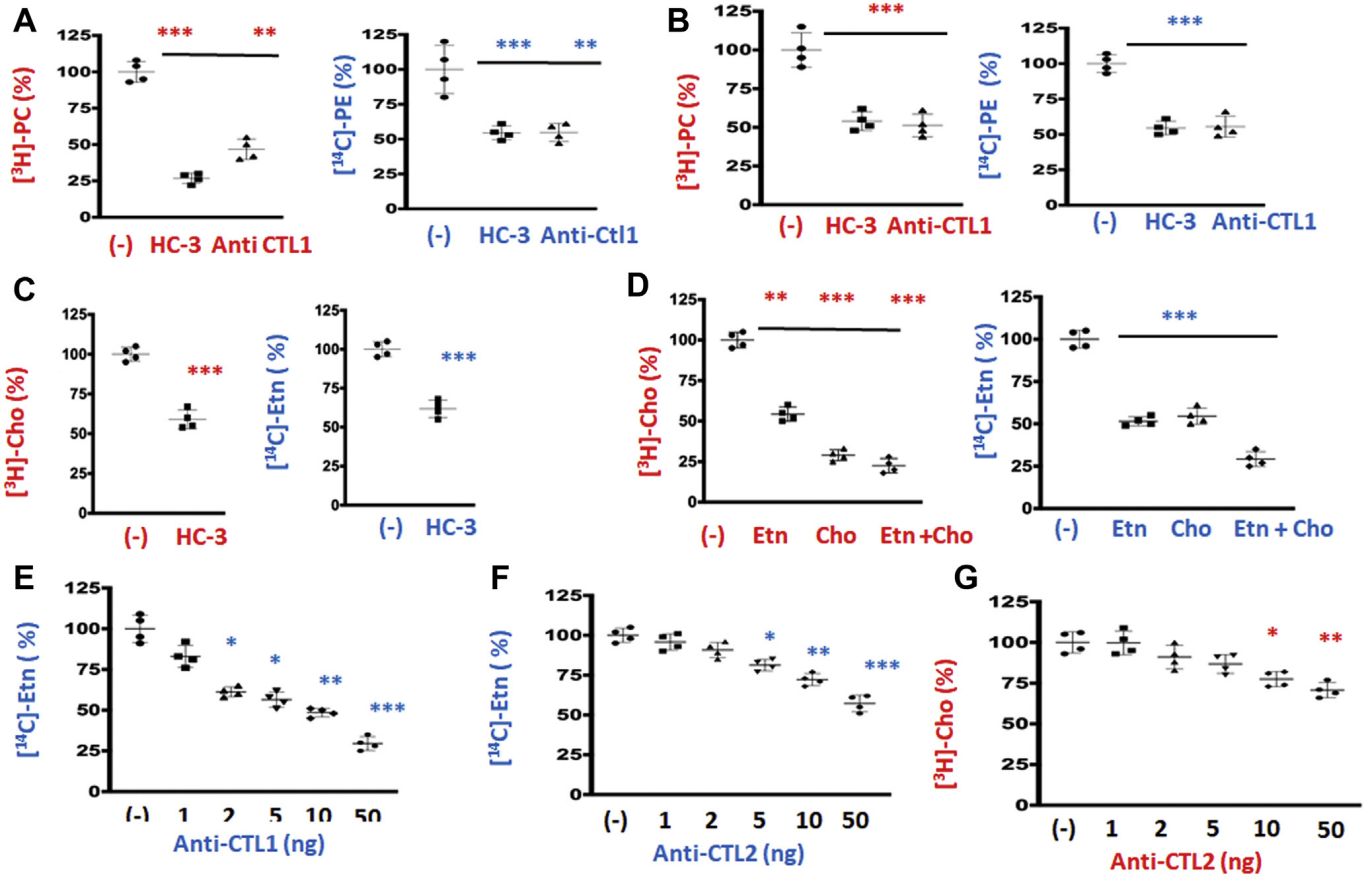
**Ethanolamine transport resembles CTL1- and CTL2-mediated choline transport.***A* and *B*, MCF-7 (*A*) and MCF-10 (*B*) cells were pretreated with CTL1/2 inhibitor hemicholinium (HC-3, 200 μM) or anti-CTL1 antibody (1:500) and then radiolabeled with 0.2 μCi [^14^C]-Etn or [^3^H]-Cho for 24 h. Both [^3^H]PC and [^14^C]PE were significantly reduced in HC-3- and anti-CTL1-treated cells relative to untreated (−) cells. *C*, the 0.2 μCi [^14^C]-Etn or [^3^H]-Cho uptake measured after 20 min in MCF7 cells was significantly reduced with HC-3 (200 μM) added 20 min prior to the labeling. *D*, separately or together, excess of “cold” Cho and Etn (200 μM each, 20 min) inhibited the transport of both radiolabeled substrates. *E*–*G*, anti-CTL1 and anti-CTL2 antibodies inhibited [^14^C]-Etn (*E* and *F*) and [^3^H]-Cho (*G*) transport in primary human fibroblasts in a dose–response manner. Each measurement represents the mean ± SD (n = 4); ∗*p* < 0.05, ∗∗*p* < 0.01, ∗∗∗*p* < 0.001.

To assess the magnitude by which CTL1/2 inhibition affects PE and PC levels, two types of cells were characterized for phospholipid metabolism (MCF-7 and MCF-10) (16, 17). The cells were treated for 24 h with [^3^H]-glycerol, to label the entire glycerolipid pools (steady-state levels) in the presence and absence of CTL1 transport inhibitor hemicholinium-3 (HC-3) or CTL1 specific antibody (Fig. 1, *A* and *B*). It is surprising that HC-3 reduced the steady-state levels not only of PC (25%–50%) but also of PE (50%) in both cell types. CTL1 antibody similarly reduced PC and PE levels (40%–50%), further indicating that CTL1 could be involved in the transport of Etn, in addition to its well-characterized function in Cho transport (15, 18, 19). [^3^H]-Cho and [^14^C]-Etn transport were similarly inhibited with the CTL1 inhibitor HC-3 (Fig. 1*C*), and they compete for the same transport system (Fig. 1*D*). [^14^C]-Etn and [^3^H]-Cho transports were similarly diminished when equal, 200 μM, Etn, Cho, and Etn + Cho were applied, respectively (Fig. 1*D*). Furthermore, CTL1 antibody inhibited ^14^C-Etn transport in a concentration-dependent manner with an IC_50_ of 50 ng (Fig. 1*E*). CTL2 antibody also inhibited both ^14^C-Etn and ^3^H-Cho transports in a concentration-dependent manner with LC_50_ 50 ng (Fig. 1, *F* and *G*). Together, the data showed that Etn is a substrate for CTL1- and CTL2-mediated transports, in addition to already known functions in Cho transport.

### CTL1 is a high-affinity and CTL2 is a low-affinity Etn transporter

**Figure 2:**
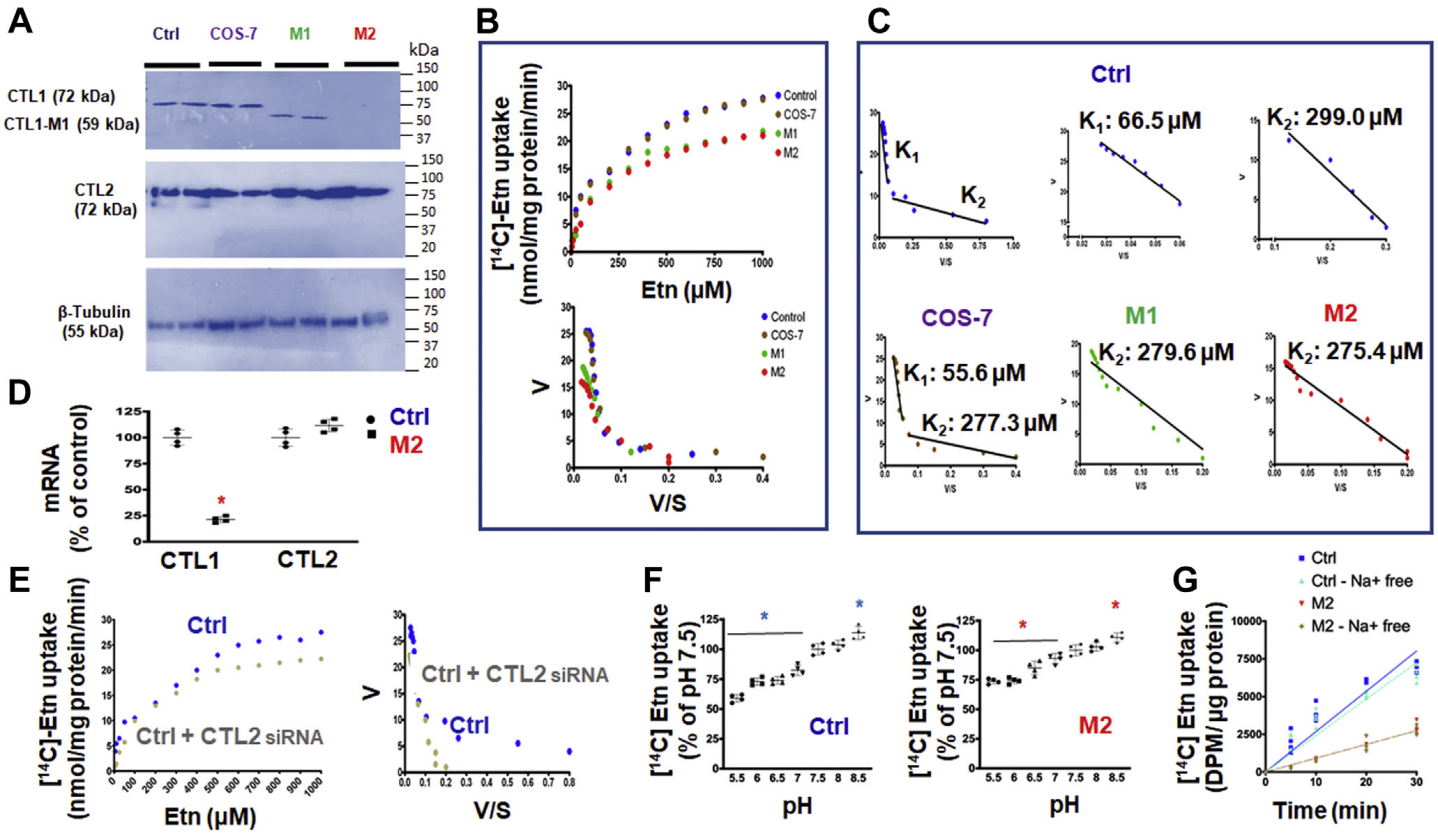
**Characteristics of low- and high-affinity Etn transports.***A*, CTL1 protein (72 kDa) was detected in control human fibroblasts (Ctrl) and monkey COS-7 cells but not in CTL1-deficient human fibroblasts (M1 and M2). An intact CTL2 (72 kDa) protein and β-tubulin (55 kDa) control were detected in all cells. *B*, saturation curves of Etn transport (V [velocity] *versus* S [substrate concentration]) were produced by measuring the uptake of [^14^C]-Etn (0–1000 μM, 20 min) in Ctrl, M1, M2, and COS-7 cells. *C*, the Eadie–Hofstee plots derived from those curves demonstrated the presence of two Etn transport systems, with binding constants K1 (higher affinity) and K2 (lower affinity), in Ctrl and COS-7 cells. Only one transport system with the lower affinity was present in CTL1-deficient M1 (279.6 ± 17.1 μM [K_2_]) and M2 (275.4 ± 13.9 μM [K_2_]) cells. *D*, CTL2 expression was not affected, whereas CTL1 mRNA was almost diminished in M2 cells. *E*, saturation curves and Eadie–Hofstee plots of siRNA-CTL2–treated Ctrl cells showed the presence of a single Etn transport of a higher affinity (CTL1-mediated, K1, transport); the low-affinity, CTL2-mediated K2 transport was specifically depleted with the siRNA-CTL2 treatment. *F*, the [^14^C]-Etn uptake is downregulated with low extracellular pH (high [H^+^]) and upregulated at high pH (low [H^+^]) in both Ctrl (CTL1+CTL2) and M2 (CTL2 only) cells. *G*, time course (0–30 min) of 10 μM [^14^C]-Etn uptake in the presence and absence of Na^+^ ions in Ctrl and M2 cells. Each point represents the mean ± SD (n = 4), ∗*p* < 0.05.

We studied the kinetics of Etn transport in monkey COS-7 cells and control (Ctrl) and CTL1 deficient (M1=*SLC44A1*^ΔAsp517^ and M2=*SLC44A1*^Ser126^) primary human fibroblasts. As expected, COS-7 cells and Ctrl fibroblasts expressed CTL1 and CTL2 proteins, whereas CTL1 mutant fibroblasts M1 and M2 only expressed CTL2 protein (Fig. 2*A*). ^14^C-Etn transport rates (V) plotted against [Etn] produced a series of saturation curves, as expected for protein-mediated transports (Fig. 2*B*). *V*_*max*_ values were nearly identical in Ctrl and COS-7 cells (*V*_*max*_ *=* 26.9 and 26.3 nmol/mg protein/min) and CTL-deficient M1 and M2 cells had reduced but similar *V*_*max*_ = 20.6 to 21.2 nmol/mg protein/min (Fig. 2*B*), apparently caused by the absence of the CTL1 transport component. Indeed, the Eadie–Hofstee plots derived from the saturation curves were biphasic in Ctrl fibroblasts and COS-7 cells and linear for M1 and M2 cells (Fig. 2*C*). This type of behavior indicated the presence of two distinct transport systems in Ctrl and COS-7 cells with two binding constants, of high and low affinity for Etn, and one transport system of a lower affinity in M1 and M2 cells. As further shown in Figure 2*C*, Ctrl fibroblasts, high-affinity (K_1_ = 66.5 ± 8.5 μM) and low-affinity (K_2_ = 299.0 ± 13.1 μM) Etn bindings were similar to COS-7 cells bindings (K_1_ = 55.6 ± 14.8 μM and K_2_ = 277.3 ± 7.9 μM). On the other hand, CTL1-deficient M1 and M2 cells are characterized by a single transport with a binding constant for Etn of 275.4 to 279.6 μM, which is the second (K_2_), low-affinity, binding constant as determined in Ctrl and COS-7 cells (Fig. 2*C*). M1 and M2 cells only express CTL2 and at levels similar to Ctrl and Cos 7 cells, and do not have a functional CTL1 protein (Fig. 2, *A* and *D*), strongly implicating CTL2 as responsible for the low-affinity Etn transport. Indeed, CTL2 depletion by siRNA knockdown in Ctrl cells abolished the low-affinity transport component while the high-affinity component remained intact (Fig. 2*E*). This analysis also confirmed that the high-affinity transport (K_1_), which is absent in M1 and M2 cells and remained intact in CTL2 siRNA-treated Ctrl, is CTL1-mediated Etn transport.

Since the effects of pH and [Na^+^] ions on choline transport are well established (19), their effects on Etn transport were also investigated (Fig. 2, *F* and *G*). Etn transport in Ctrl (CTL1 + CTL2 transport) and CTL1-deficient M2 fibroblasts (CTL2 transport) (Fig. 2*F*) was reduced when extracellular pH was lowered from 7 to 5.5 and stimulated when pH was increased to 8.5. In addition (Fig. 2*G*), as expected, the rate of Etn transport in Ctrl cells was higher than in M2 cells but the rates were not modified when Na^+^ ions were replaced by Li^+^ ions in the uptake buffer. Altogether, the data established that CTL1 and CTL2 acts as Etn/H^+^ antiporters, driven by a proton gradient, and they are both independent of Na^+^, as in case of Cho transport (19).

### CDP-Etn Kennedy pathway is downregulated by CTL1 deficiency

**Figure 3:**
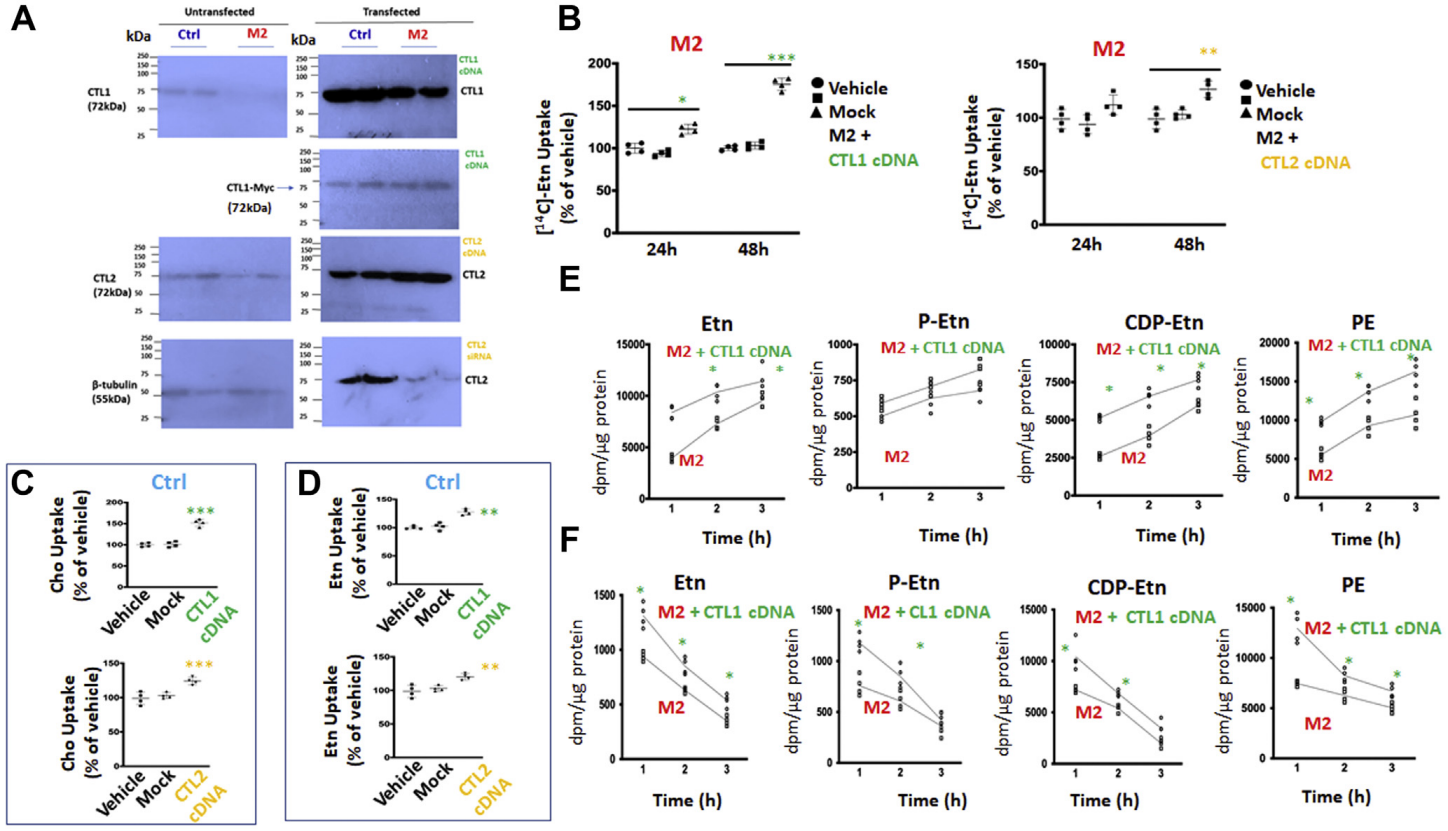
**Contributions of CTL2 to PE synthesis by the CDP-Etn Kennedy pathway.***A*, [^14^C]-Etn 1 to 3 h radiolabeling of CDP-Etn Kennedy pathway intermediates (Etn, P-Etn, and CDP-Etn) and PE synthesis in Ctrl and CTL1-deficient M2 cells. *B*, pulse-chase [^14^C]-Etn labelling of the CDP-Etn pathway intermediates and PE turnover in Ctrl and M2 cells. *C* and *D*, [^3^H]Glycerol labelling of glycerolipid pools showing how PC was unchanged, while PE and PS were decreased in M2 cells relative to Ctrl cells (*C*) and how TAG levels were increased, whereas DAG was decreased in M2 cells (*D*). *E*, Pcyt2 activity was significantly reduced in M2 cells. *F*, Pcyt1 mRNA expression was decreased by 65% in M2 cells, while Pcyt2, CK, and EK mRNA expression were decreased by 20% in M2 cells. Each point represents the mean ± SD (n = 4), ∗ *p* < 0.05, *∗∗ p* < 0.01, *∗∗∗ p* < 0.001.

Since this is the first time that Etn transporters are identified, it is important to establish if they are functionally linked to the CDP-Etn Kennedy pathway for PE synthesis. We performed pulse (synthesis) and pulse-chase (degradation) radiolabeling with [^14^C]Etn in Ctrl and CTL1-deficient M2 cells, to establish the contributions of total and CTL2 transport to the CDP-Etn Kennedy pathway (Fig. 3, *A* and *B*). As shown in (Fig. 3*A*), the total incorporation of [^14^C]Etn and the rates of synthesis (slopes) of the pathway intermediates phospho-Etn (P-Etn) and CDP-Etn and the final product PE were lower in M2 cells than in Ctrl cells. Similarly reduced was the [^14^C]Etn incorporation and P-Etn and CDP-Etn disappearance in M2 cells in the pulse-chase experiments (Fig. 3*B*). In addition, the [^3^H]glycerol radiolabeling of glycerolipid equilibrium pools (the steady-state levels) showed unchanged PC; reduced PE, PS, and DAG; and increased triglycerides (TAGs) in M2 cells (Fig. 3, *C* and *D*). Therefore, reduced Etn transport and slower P-Etn and CDP-Etn formation collectively slowed the CDP-Etn pathway (Fig. 3, *A* and *B*) and reduced PE levels (Fig. 3*C*) in M2 cells. Reduced DAG and increased TAG in M2 cells indicate DAG switching toward TAG synthesis. DAG is a common substrate for phospholipids and TAG, and under conditions of reduced PE synthesis by the CDP-ethanolamine Kennedy pathway (Fig. 3, *A* and *B*) the majority of DAG is directed toward TAG synthesis (Fig. 3*D*), as established in three CDP-ethanolamine Kennedy pathway knockout mouse models (20, 21, 22). In addition to reduced DAG and increased TAG, phosphatidic acid, phosphatidylglycerol, and phosphatidylinositol were also increased in CTL1-deficient cells (15), implying an upregulation of the CDP-DAG pathway (23, 24) when Etn transport and CDP-Eth Kennedy pathway are impaired.

The CDP-Etn formation from PEtn is usually the rate-regulatory step in the Kennedy pathway and is controlled by Pcyt2 (CTP:phosphoethanolamine cytidylyltransferase) (11). Indeed, in accordance with reduced CDP-Etn formation above, the activity and expression of Pcyt2 were also reduced in M2 cells (Fig. 3, *E* and *F*). The expression of Etn kinase (EK) was similarly decreased by 25% (Fig. 3*F*), explaining why the formation of P-Etn was reduced in M2 cells (Fig. 3, *A* and *B*). In addition to PE levels, PS levels were reduced in M2 cells, but the expression of PS synthesis (PS syntase1/2-PSS1/2) and PS degradation (PS decarboxylase-PSD) genes (Fig. 3*F*) was unaltered (Fig. 3*C*). The expression of the PC synthesis genes Pcyt1 (CTP:phosphocholine cytidylyltransferase) was decreased by 70% and that of choline kinase (CK) by 25% in M2 cells (Fig. 3*F*), yet unexpectedly PC levels were unchanged (Fig. 3*C*). We previously established (15) that the constant PC levels in CTL1-deficient M1 and M2 cells are maintained by reduced PC turnover and increased formation from other phospholipids (PC is made at the expanse of PE and PS), as the main mechanism to maintain PC as a source of choline in a new neurodegenerative disorder caused by frame-shift mutations in the CTL1 gene M1=*SLC44A1*^ΔAsp517^ and M2=*SLC44A21*^Ser126^ (15). These data collectively provided strong genetic and metabolic evidence that CTL1 and CTL2 independently contribute not only to the CDP-Cho but also to the CDP-Etn Kennedy pathway. CTL2 is not overexpressed in deficient M1 and M2 cells (Fig. 2*A*), and as such it cannot compensate for the absence of CTL1 in those cells and affected individuals (15).

### Overexpressed CTL1 and CTL2 participate in choline and ethanolamine transport

**Figure 4:**
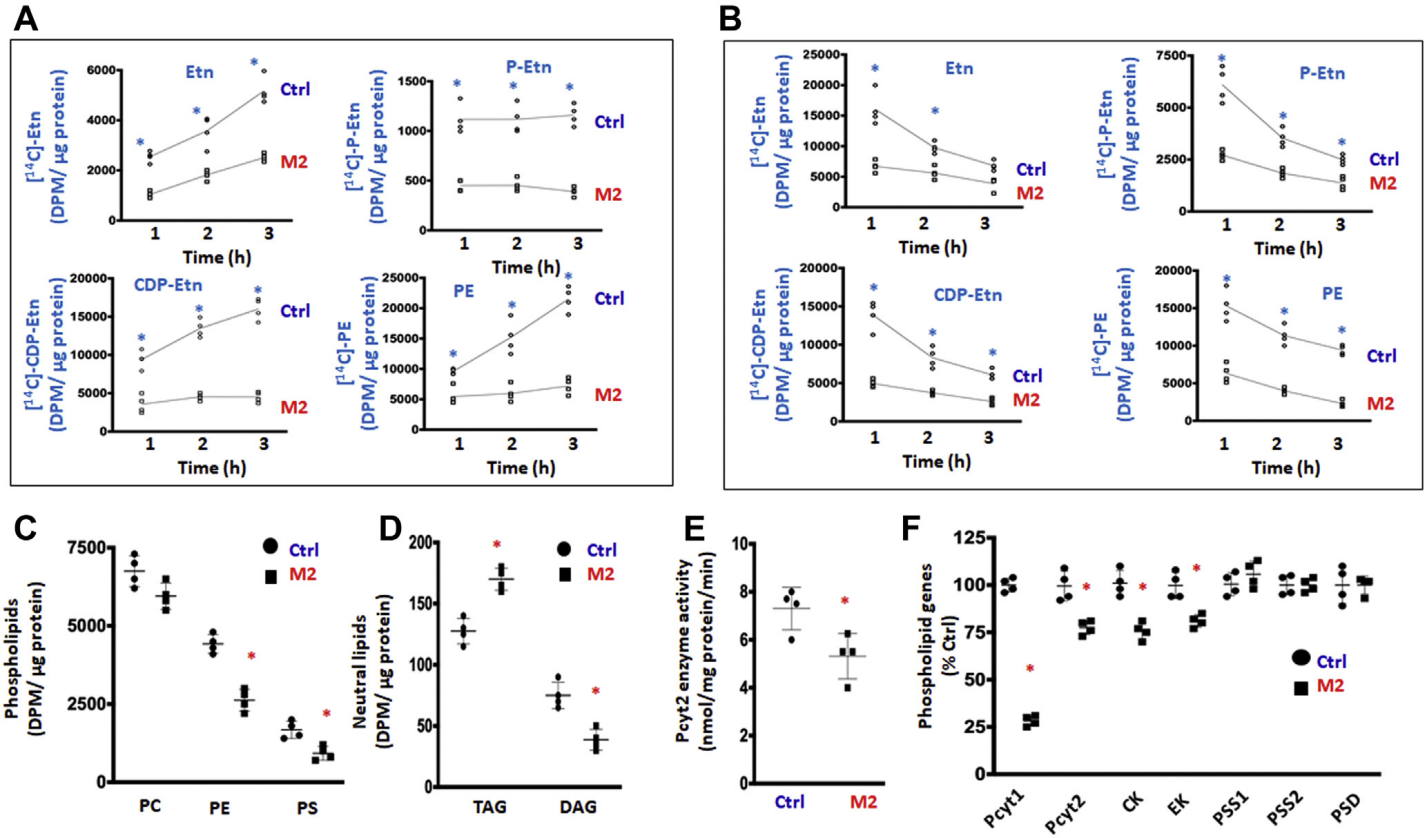
**CDP-Etn Kennedy pathway is regulated with the levels of CTL1 and CTL2 expression.***A*, CTL1 and CTL2 protein levels in Ctrl and CTL1-deficient M2 cells transfected with CTL1 cDNA, CTL2 cDNA, and CTL2 siRNA. *B*, in M2 cells transfected with CTL1 cDNA, Etn uptake was increased by 25% after 24 h and 75% after 48 h. In M2 cells transfected with CTL2 cDNA, Etn uptake increased by 25% after 48 h. *C*, Cho uptake at 48 h was increased by 50% in Ctrl cells transfected with CTL1 cDNA and 25% in Ctrl cells transfected with CTL2 cDNA. *D*, Etn uptake was increased by 25% in Ctrl cells transfected with CTL1 cDNA and by 25% in Ctrl cells transfected with CTL2 cDNA. *E*, [^14^C]-Etn radiolabeling of CDP-Etn pathway and PE synthesis in CTL1-overexpressing M2 cells. *F*, [^14^C]-Etn 1 to 3 h pulse-chase analysis of the CDP-Etn pathway and PE degradation in CTL1-overexpressing M2 cells. Vehicle, untransfected cells; Mock, cells transfected with empty vector. Each point represents the mean ± SD (n = 4), ∗*p* < 0.05, *∗∗p* < 0.01*,* ∗∗∗*p* < 0.001.

To demonstrate that CTL1 and CTL2 are both Etn and Cho transporters, the cells were transiently transfected with CTL1 cDNA or CTL2 cDNA and the protein expression and transport determined after 48 h. M2 cells lack endogenous CTL1 protein, as demonstrated in the left panel in Figure 4*A*. CTL1 cDNA increased CTL1 protein in both cells with a higher abundance in transfected Ctrl than in M2. In the transfected cells, the CTL1 antibody detects total CTL1 protein, from endogenously present CTL1 and plasmid DNA; Myc antibody on the other hand detects only transfected CTL1-Myc in similar levels since equal amounts of plasmid DNA were used in transfections (Fig. 4*A*-*right panel*).

Ctrl and M2 cells already have endogenous CTL2, and transfection with CTL2 cDNA further increased the CTL2 levels. On the other hand, when cells were treated with CTL2 siRNA, the treatment reduced CTL2 protein and diminished the low-affinity Etn transport described in Figure 2*E*. Accordingly, CTL1 cDNA increased Etn transport by 100% and CTL2 cDNA increased Etn transport 25% in M2 cells 48 h post transfection (Fig. 4*B*). In Ctrl cells, CTL1 cDNA increased Cho (Fig. 4*C*, *upper panel*) and Etn (Fig. 4*D*, *upper panel*) transport by 50% and 25%, respectively. CTL2 cDNA similarly increased Cho and Etn transport by 25% (Fig. 4, *C* and *D*-*lower panels*). Overall, the transfection data corroborated the kinetic data in Figures 1 and 2 and showed that CTL1 and CTL2 promote transport with similar affinities for Cho and Etn, further showing their functional connection with both arms of the Kennedy pathway.

### Overexpressed CTL1 facilitates CDP-Etn Kennedy pathway in deficient cells

To establish if the overexpression of CTL1 in mutant cells can facilitate the CDP-Etn Kennedy pathway, CTL1 cDNA-transfected (M2+CTL1) and CTL1-deficient (M2) cells were monitored with [^14^C]Etn radiolabeling in pulse (Fig. 4*E*) and pulse-chase (Fig. 4*F*) experiments. As expected, the incorporation of the [^14^C] in Etn, CDP-Etn, and PE was significantly increased in CTL1 cDNA-transfected relative to untransfected M2 cells. Both types of labelling experiments showed increased P-Etn and CDP-Etn degradation and increased PE synthesis and turnover in M2 transfected cells. Overall, these data demonstrated that the stimulated Etn transport by CTL1 expression increased the flux through the CDP-Etn pathway in CTL1-deficient M2 cells.

### Pharmacological and siRNA inhibition of Etn transport

**Figure 5:**
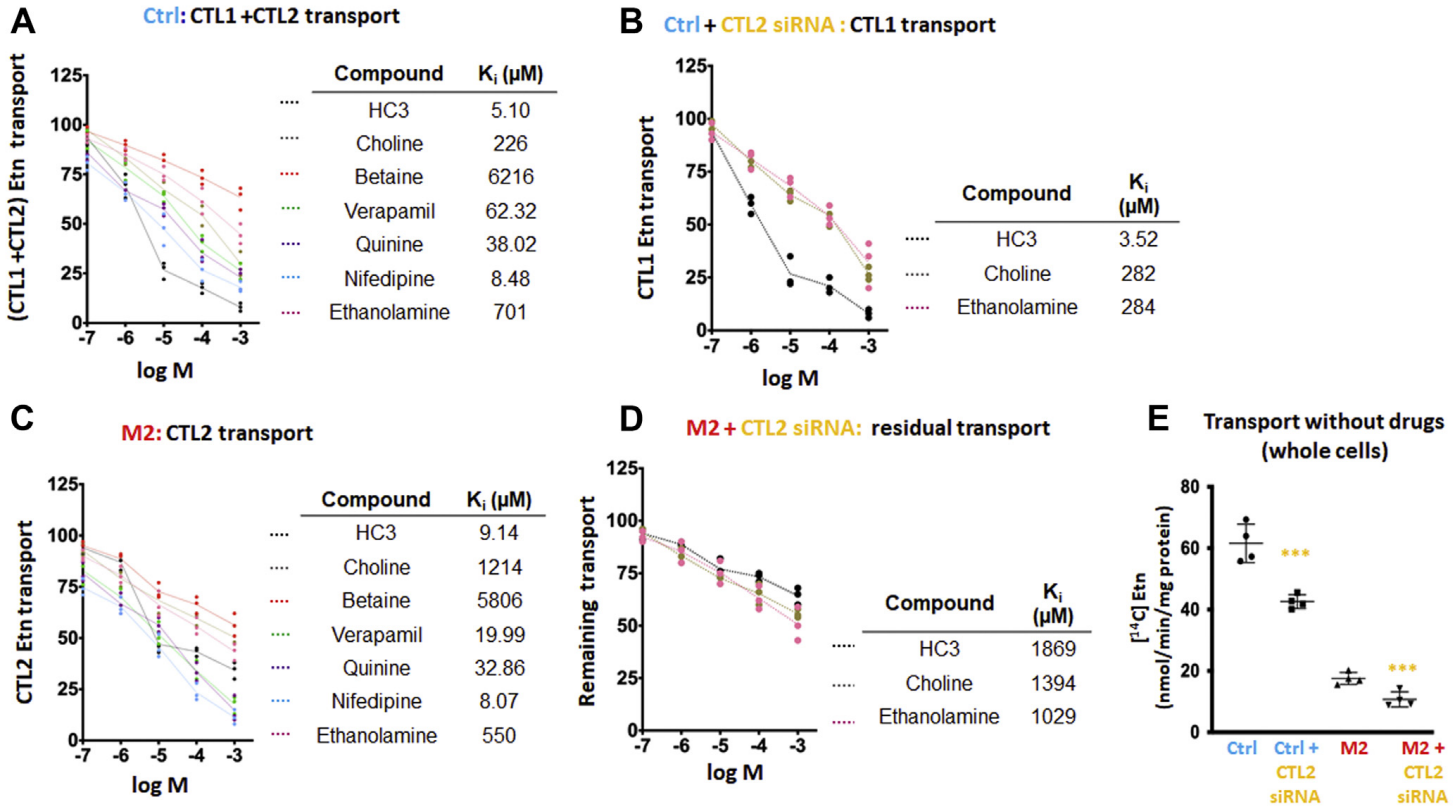
**Pharmacological distinction of ethanolamine transport and transporters.***A*, semi-log plots of Etn transport inhibition in Ctrl cells and (*B*) Ctrl cells transfected with CTL2 siRNA. *C*, semi-log plots of Etn transport inhibition in M2 cells and (*D*) M2 cells transfected with CTL2 siRNA. The cells were preincubated with test compounds for 30 min, and the uptake of 20 μM [^14^C]-Etn was measured for 20 min. The obtained Ki inhibitory constants and contributing transports for all cell types and treatments are indicated. *E*, [^14^C]-Etn uptake in the absence of inhibitors in Ctrl and M2 cells with and without siRNA transfection. Each point represents the mean ± SD (n = 4); ∗∗∗*p* < 0.001.

It is well known that various organic cations can inhibit CTL1-mediated Cho transport (25). We assessed the inhibitory effect of organic cations and CTL2 knockdown on Etn transport in Ctrl and M2 cells (Fig. 5). This helped us understand the magnitude at which CTL1 and CTL2 contributed to Etn transport. Total (CTL1+CTL2) transport present in Ctrl cells (Fig. 5*A*), CTL1-mediated transport in Ctrl treated with CTL2 siRNA (Fig. 5*B*), CTL2-only transport in M2 cells (Fig. 5*C*), and residual, CTL1- and CTL2-independent transport present in CTL2 siRNA-treated M2 cells (Fig. 5*D*) were quantified. In each case, the inhibitory constants *K*_*i*_ were deduced from semi-log plots (% of remaining Etn transport *versus* logM concentration) and compared between different transport conditions.

As expected, the CTL1- and CTL2-specific inhibitor HC-3 strongly inhibited Etn transport with *K*_*i*_ values of 3.52 μM (CTL1), 9.14 μM (CTL2), and 5.10 μM for the total (CTL1 + CTL2) transport. Nifedipine (a calcium channel blocker) was as potent as HC-3 with *K*_*i*_ = 8.07 to 8.48 μM. Verapamil (a calcium channel blocker) was an intermediate CTL1/2 inhibitor with *K*_*i*_ = 20 to 62 μM. In addition, quinine (antimalaria drug) with *K*_*i*_ = 33 to 38 μM was a medium CTL1/2 inhibitor, whereas the Cho oxidation product betaine was a poor inhibitor (*K*_*i*_ = 5806–6216 μM) of CTL1 and CTL2 Etn transports (Fig. 5, *B* and *C*).

The inhibition of CTL1-specific transport (Fig. 5*B*) with excess choline and Etn was similar, with *K*_*i*_ = 282 to 284 μM. The *K*_*i*_, however, differed for CTL2-specific transport (Fig. 5*C*), with *K*_*i*_ = 1214 μM for Cho and 550 μM for Etn. The *K*_*i*_ value for the total (CTL1 + CTL2) transport (Fig. 5*A*) was 226 μM for Cho and 701 μM for Etn. Thus, the *K*_*i*_ values indicated that Cho and Etn are transported similarly by the high-affinity transporter CTL1. The low-affinity transporter CTL2, however, had reduced and different affinity for Cho and Etn, with more preference for Etn as its substrate. Residual Etn transport (unrelated to CTL1 and CTL2) was distinguished by CTL2 siRNA treatment of M2 cells (Fig. 5*D*). This residual transport component has a low affinity for Cho and Etn (*K*_*i*_ = 1029–1394 μM) and three orders of magnitude higher *K*_*i*_ = 1869 μM for HC-3 showing that is not a CTL-related transport. Finally, comparison of all transport velocities (Fig. 5*E*) showed a general order of contributions, from the high-affinity CTL1 (Ctrl + CTL2 siRNA), low-affinity CTL2 (M2), and the residual very-low-affinity (M2+CTL2 siRNA) transports for Etn. CTL1(Ctrl) contributed 80% and CTL2 (M2) 12.5%, and the unrelated residual transport (M2 + CTL2 siRNA) accounted for 7.5% to the total transport. The Ctrl and M2 cells express various unspecific transporters that could be contributing to this residual transport (15).

### CTL1 and CTL2 mediate Etn transport to mitochondria

**Figure 6:**
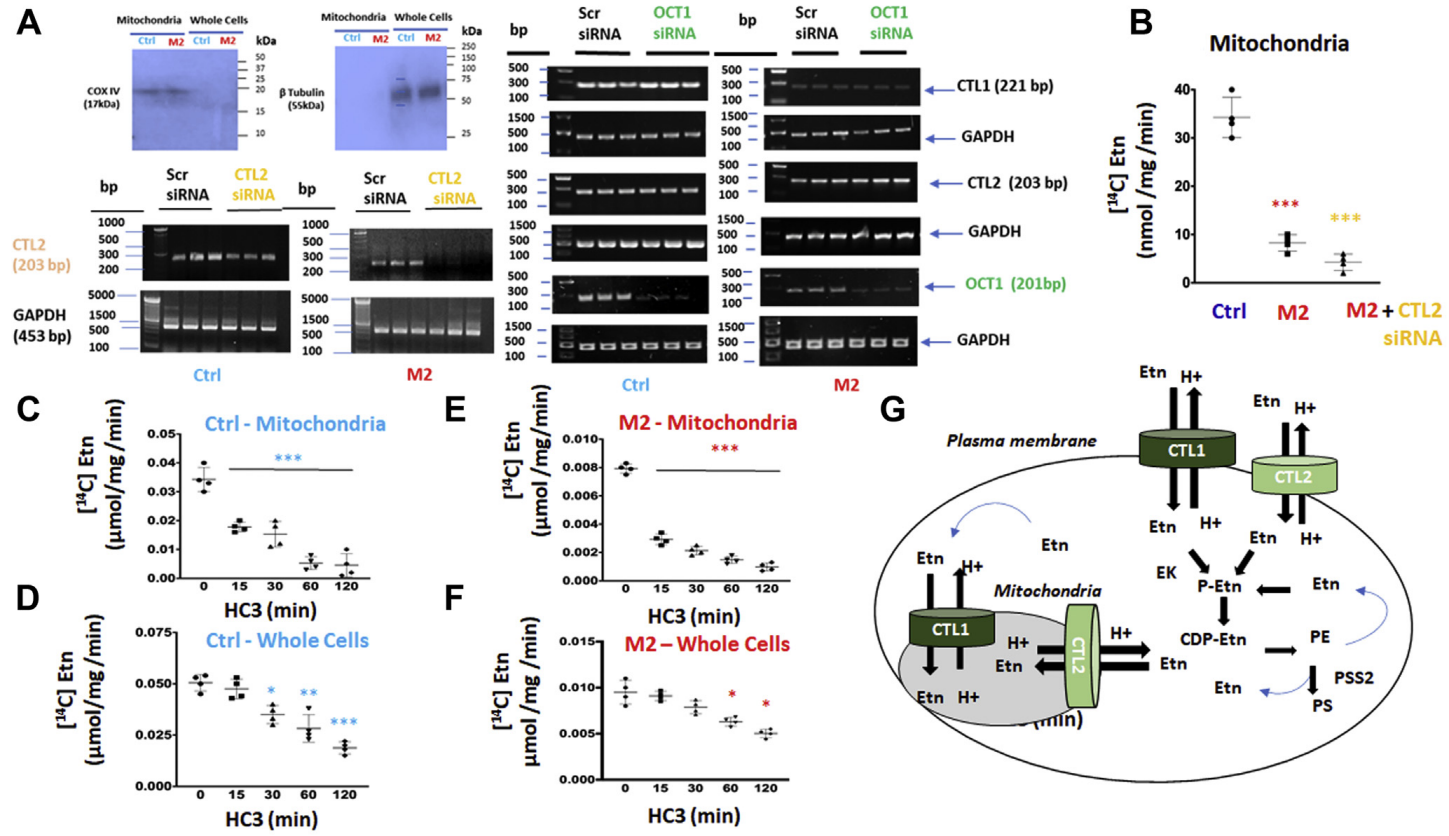
**Characteristics of mitochondrial ethanolamine transport.***A*, CTL1, CTL2, and Oct1 mRNA expression in siRNA-Oct1 and siRNA-CTL2 treated and untreated Ctrl and M2 cells. Western blots of cytochrome c oxidase subunit IV (COXIV) mitochondrial marker demonstrating purity of Ctrl and M2 mitochondria. *B*, mitochondrial Etn transport and the effect of CTL2 knockdown in M2 cells; Etn uptake in M2 mitochondria was reduced to 25% of Ctrl mitochondria and further diminished with siRNA depletion of CTL2 in M2 cells. Time course inhibition of Etn uptake in (*C*) Ctrl mitochondria and (*D*) whole cells treated with 20 μM HC-3. Time course of Etn uptake in (*E*) M2 mitochondria and (*F*) M2 cells treated with 20 μM HC-3. Each point represents the mean ± SD (n = 4), ∗ *p* < 0.05, ∗∗ *p* < 0.01, ∗∗∗*p* < 0.001. *G*, schematic representation of ethanolamine transport mechanisms at the cell surface and mitochondria. CTL1 and CTL2 are ethanolamine/proton antiporters of high and low affinity, respectively. They are responsible for extracellular uptake and intracellular balance of [Etn]. Extracellular Etn is immediately phosphorylated by ethanolamine kinase (EK) and further consumed by the CDP-Etn pathway to form phosphatidylethanolamine (PE) phospholipid. The intracellular Etn is released by PE degradation by lipolysis and PE exchange to phosphatidylserine (PS), by PS synthase 2 (PSS2), and it can enter mitochondria by both transporters. GAPDH, glyceraldehyde 3-phosphate dehydrogenase.

CTL1 and CTL2 are present in the mitochondria (4, 5). We used cytochrome c oxidase, subunit IV (COXIV) as a marker of mitochondria isolated from Ctrl and M2 cells and CTL2 mRNA expression for the siRNA knockdown of CTL2 transport (Fig. 6*A*). Immunoblotting in Figure 6*A* (*top left panel*) indicates that COX IV was abundant in isolated mitochondria, and since the whole cells biomarker, β-tubulin, was detected only in the whole cells and not in the mitochondria, the results indicate that the mitochondrial isolate was pure from other subcellular compartments.

We used organics cation transporter 1 (OCT1) siRNA and two scrambled (Scr) siRNAs as controls for CTL2 siRNA knockdown (Fig. 6*A*). CTL2 siRNA reduced CTL2 mRNA in both Ctrl and M2 cells. As expected, OCT1 siRNA only reduced OCT1 expression and did not affect CTL1, CTL2, and GAPDH mRNA expression. (Fig. 6*A*). By comparing the contributions of all mitochondrial transport components (Fig. 6*B*), CTL1+CTL2 (Ctrl) contributed 70%, CTL2 (M2) contributed 20%, and the residual unrelated transport (M2 + CTL2 siRNA) contributed 10% to the total mitochondrial Etn transport. We also compared the rates of [^14^C]-Etn transport in the isolated mitochondria and the whole cells and in the presence and absence of the specific inhibitor HC-3. As expected, the CTL1 and CTL2 inhibitor HC-3 blunted Etn uptake in a time-dependent manner in the mitochondria (Fig. 6, *C* and *E*) and the whole cells (Fig. 6, *D* and *F*). In the absence of HC-3, the rate of Ctrl mitochondrial transport was similar to the whole cell Ctrl transport (0.04 and 0.05 μmol/mg/min, respectively); M2 mitochondrial transport was also similar to the whole cell transport (0.008 and 0.01 μmol/mg/min, respectively) (Fig. 6, *E* and *F*), demonstrating that the same proteins are responsible for the transports in the whole cell and mitochondria. In addition, the M2 mitochondrial (CTL2 only) transport was 5-fold slower than the total (CTL1+CTL2) transport of the Ctrl mitochondria. Taken together, these data established that CTL1 and CTL2 mediate mitochondrial Etn transport with the same kinetic properties as in the whole cells.

## Discussion

PE and PC are bilayer-forming phospholipids involved in fundamental membrane processes, growth, survival, and cell signaling (11). PE and PC are similarly synthesized by CDP-Etn and CDP-Cho Kennedy pathway, in which the extracellular substrates Cho and Etn are actively transported into the cell, phosphorylated, and coupled with DAGs to form the final phospholipid product. Cho and Etn released from PC and PE also need to be transported in and out of the cytosol and mitochondria or reincorporated into the Kennedy pathway. The plasma membrane CTL1 is firmly assigned to Cho transport for PC synthesis (15), yet the exact function of the mitochondrial CTL1 is still not clear. In the liver and kidney mitochondria, Cho is specifically oxidized to betaine, the major methyl donor in the one-carbon cycle (6). Since it is broadly expressed, it is proposed that the mitochondrial CTL1 could maintain the intracellular pools of Cho and as ^+^H-antiporter could regulate the electrochemical/proton gradient in the mitochondria (5, 15, 18).

We and others clearly established that CTL1 is the main choline transporter for phospholipid synthesis (3, 4). CTL2 was implicated in choline transport since it was frequently expressed in various cells, but transport characteristics of CTL2 were not clearly established. CTL3, -4, and -5 are poorly characterized members of the family, and their transport functions are not clearly established (3, 4). Based on our extensive work on CTL1 and Cho transport (3, 4, 5, 6, 7, 8, 15) and known similarities between Cho and Etn transports in various conditions (12, 13, 14) we postulated that CTL1 could be that long-searched-for Etn/Cho transporter and the last missing link between CDP-Cho and CDP-Etn pathways for phospholipids synthesis. We conducted an extensive number of kinetic, metabolic, and genetic experiments to solidify this hypothesis. We established that CTL1 mediated a high-affinity Etn transport with *K*_*1*_ = 56 to 67 μM and that CTL2 mediated a low-affinity Etn transport with *K*_*2*_ = 275 to 299 μM in primary human fibroblasts and monkey Cos7 cells. Of importance, the CTL1 affinity constant for Etn binding is in the range of physiological Etn concentration in rat and humans (10–75 μM) (13) and explains why CTL1 contributed the most (70%–80%) of the Etn transport in the whole cells and mitochondria.

We recently described the first human disorder caused by homozygous frame-shift mutations in the CTL1 gene *SLC44A1*: M1= *SLC44A1* ^ΔAsp517^, M2= *SLC44A1* ^ΔSer126^, and M3= *SLC44A1* ^ΔLys90^ (15). After an extensive characterization of transport and metabolism in patient’s fibroblasts it was apparent that diminished Cho transport is the primary cause of this new neurodegenerative disorder with elements of childhood-onset parkinsonism and MPAN (mitochondrial membrane protein–associated neurodegeneration)-like abnormalities. Paradoxically, although Cho transport and CDP-Cho Kennedy pathway were diminished, PC remained preserved in the cerebrospinal fluid and skin fibroblasts of the affected individuals (15). The cell membranes were, however, drastically remodeled and depleted of PE and PS, apparently as a homeostatic response to preserve PC and prevent Cho deficiency in the affected individuals (15). Since the majority of PE is produced *de novo* by the CDP-Etn pathway, we utilized CTL1 mutant cells to establish if Etn transport and *de novo* PE synthesis were reduced in the affected individuals. We showed that (M2) *SLC44A1* ^ΔSer126^ patient fibroblasts rely on CTL2 for Cho and Etn transport and were devoid of CTL1-mediated Etn transport, which revealed a new biological role for CTL1 and CTL2 that could help in developing new treatment strategies for this devastating disease.

Cho supplementation led the membrane lipids and organelle recovery in CTL1 mutant fibroblast (15) suggesting that transporters other than CTL1 could support the transport when extra substrate became available. We focused on CTL2 as the most plausible alternative candidate for Cho and Etn transport in CTL1-deficient cells (19, 25, 26, 27). We separated CTL2 and CTL1 transports using specific antibodies and depletion and overexpression strategies in CTL1 M2 mutant and Ctrl cells. As a low-affinity Etn transporter CTL2 contributed 20% to 30% to the total transport in the whole cells and isolated mitochondria. CTL1-deficient cells had reduced but not absent CDP-Cho Kennedy pathway (15) and also had reduced CDP-Etn Kennedy pathway showing that CTL2 is able to channel both substrates for phospholipid synthesis, albeit with a reduced capacity. Apparently, in the individuals with the *SLC44A1* homozygous mutation (15) under normal physiological conditions CTL2 did not compensate the complete loss of CTL1 but as a low-affinity (high-capacity) transporter it could be beneficial in delivering extra Cho and Etn.

PE plays an important structural role in mitochondria (28, 29, 30) and is important for stabilizing the electron transport complexes (31, 32, 33, 34, 35). Both CTL1 and CTL2 are prominently present in mitochondria (3, 19). When identical proteins are involved, it is expected that transport characteristics are similar even in separate cellular compartments; this was the case with Etn transport in the whole cells and mitochondria of Ctrl cells (40–50 nmol/mg/min; CTL1+CTL2) and M2 whole cells and mitochondria (8–10 nmol/mg/min CTL2 only). We clearly demonstrated that CTL1 and CTL2 channel the extracellular Etn into the Kennedy pathway. Why there is a cellular need for active Etn transport and involvement of the low- and high-affinity transporters in the mitochondria is not clear. Mitochondrial CTL1 and CTL2 could maintain the intracellular pools of Cho and Etn, and since they are both proton antiporters, they could be significant regulators of the proton gradient in the mitochondria. Recent studies in CTL2 knockout mice established that CTL2-mediated mitochondrial Cho transport is critical for ATP and reactive oxygen species production, platelet activation, and thrombosis (36). CTL2 gene *SLC44A2* is well-established the human neutrophil antigen (37), and genetic risk factor for hearing loss, Meniere’s disease, and venous thrombosis (38). Neutrophil CTL2 could interact directly with platelets’ integrin α_IIb_β_3_ and induce neutrophil extracellular trap (NETosis) that promotes thrombosis (39). *SLC44A2* knockout mouse is protected against venous thrombosis showing that CTL2 could be an important therapeutic target for the disease (40, 41, 42). Our investigation provides insights into the novel function of CTL2/SLC44A2 as an Etn transporter, which will contribute to a better understanding of previous studies and the optimization of prevention and treatment strategies in those various diseases.

Altogether, CTL1 and CTL2 are the physiological Etn transporters in the whole cells and mitochondria, with intrinsic roles in *de novo* PE synthesis by the CDP-Etn Kennedy pathway and intracellular compartmentation of Etn. The existence of high- and low-affinity transporters is a natural strategy to cope with fluctuations with ethanolamine availability. The high-affinity CTL1 transport indicates that a relatively low concentration of ethanolamine is adequate for strong interaction with the CTL1 protein to execute ethanolamine transport. Because of a weaker interaction with ethanolamine, the low-affinity CTL2 requires a higher concentration of ethanolamine to trigger a similar physiological response. A schematic representation of Etn transport mechanisms at the cell surface and mitochondria is in Figure 6*G*. CTL1 and CTL2 are ethanolamine/proton antiporters responsible for extracellular uptake and intracellular balance of Etn. Extracellular Etn is immediately phosphorylated by EK and further consumed by the CDP-Etn pathway to form the membrane phospholipid PE. The intracellular Etn is released after PE degradation by lipolysis and/or replacement of the Etn headgroup with serine in PE to form PS by PS synthase 2 (PSS2). The metabolically released Etn can be removed, or it can enter mitochondria and/or be phosphorylated and recycled by the CDP-Etn Kennedy pathway.

## Experimental procedures

### Maintenance of cell lines

Control, M1, and M2 primary human skin fibroblasts were maintained in minimum essential media (Fisher Scientific) supplemented with 20% fetal bovine serum and 2% penicillin/streptomycin. Cells were kept in a humidified atmosphere at 37 °C and 5% CO_2_. MCF-7 human breast cancer cells, MCF-10 human mammary epithelial cells, and COS-7 fibroblast-like monkey cells were maintained in Dulbecco's modified Eagle's medium (Fisher Scientific) supplemented with 10% fetal bovine serum and 2% penicillin/streptomycin.

### Transport studies

According to our previously standardized protocols (3) cells were incubated with 0.2 μCi [^14^C]-Etn or [^3^H]-Cho with the compound of interest for 20 min and room temperature. To stop the transport, cells were washed in ice-cold KRH buffer containing 500 μM “cold” Cho or Etn. Cells were then lysed in 500 μl ice-cold lysis buffer (10 mM Tris-HCl, 1 mM EDTA, and 10 mM NaF), and the radiolabeled Cho and Etn were analyzed by LSC. Kinetic constants for Etn transport were determined as before for Cho (38). Cells were incubated (20 min) with increasing concentrations of unlabeled Etn (0–1000 μM) before being treated with 0.2 μCi [^14^C]-Etn for 20 min. The saturation curves of [^14^C]-Etn transport velocity (V) *versus* Etn concentration (S) were produced in different cell types, and the transport affinity constants (K1 and K2) were derived from linearized Eadie–Hofstee plots using GraphPad Prism software (GraphPad, Inc). To study the effect of pH on Etn uptake, the cells were treated with KRH buffers of varying pH (pH 5.5–8.5). Buffers were prepared by mixing 10 mM (N-morpholino) ethanesulfonic acid (pH 5.5) and 10 mM bicine (pH 8.5). To assess the effects of [Na^+^] on Etn uptake, the cells were subjected to either standard KRH buffer or KRH buffer with LiCl instead of NaCl. For transport inhibition, 8.0 × 10^4^ cells/well were treated with various compounds for 24 h. The concentration curves for a specific compound (HC3, Eth, Cho, betaine, verapamil, quinine, nifedipine) were produced for each cell type, and *Ki* derived from the semi log plots of transport inhibition (%) *versus* log [drug concentration] were compared.

In studies with CTL1 and CTL2 antibodies 8.0 × 10^4^ cells/well were seeded in 6-well plates and grown for 24 h. After 24 h of growth, various amounts of antibodies were added to cells and incubated for 24 h and Cho and Etn uptake were then conducted.

### Radiolabeling of the CDP-Etn Kennedy pathway and phospholipids

To analyze the CDP-Etn Kennedy pathways cells were radiolabeled with 0.2 μCi [^14^C]-Etn (ARC) for 1 to 3 h (pulse and pulse chase). Water-soluble pathway intermediates (Etn, P-Etn, and CDP-Etn) and PE were extracted and separated with the method of Bligh and Dyer (43). For pulse experiments, cells were incubated for 1, 2, and 3 h with 0.2 μCi [^14^C]-Etn, and for pulse-chase experiments, cells were incubated for 1 h with 0.2 μCi [^14^C]-Etn, washed with PBS, and then chased for 1 to 3 h with an excess of unlabeled Etn. At each time point, radiolabeled compounds were extracted with the method of Bligh and Byer and separated by TLC using appropriate standards, and radioactivity was (dpm) determined by liquid scintillation counting (20). Phospholipid (PC, PS, and PE) and neutral lipid (DAG and TAG) pools were determined by 24 h steady-state (equilibrium) radiolabeling with ^3^H-glycerol as described (20).

### Pcyt2 activity assay

The assay was conducted as described (44). In brief, cells were cultured for 24 h and 50 μg protein was dissolved in Pcyt2 reaction mixture. The mixture was treated for 15 min with 0.2 μCi [^14^C]-PEtn, and the reaction was terminated by boiling for 2 min. The radiolabeled Pcyt2 reaction product [^14^C]-CDP-Etn was isolated by TLC, and Pcyt2 activity was expressed as nmol/min/mg protein.

### RNA extraction and RT-PCR

Total RNA was isolated with TRIzol (Invitrogen, Life Technologies Incorporated). DNase I was used to eliminate genomic DNA, and cDNA was synthesized from 2 μg RNA with RNA SuperScript III Reverse Transcriptase (Invitrogen, Life Technologies Incorporated). Expression of CTL1, CTL2, CK, EK, Pcyt1, Pcyt2, PSD, PSS1, and PSS2 was determined by PCR using the primers and conditions as before (15). Reactions were standardized by amplifying glyceraldehyde 3-phosphate dehydrogenase, and the relative band intensity was quantified using ImageJ software (NIH).

### Expression of rat CTL1-Myc cDNA, murine CTL2 cDNA, and CTL2 and Oct1 siRNA

Confluent cells were transfected with 5 μg of pCMV3-ORF-C-Myc-SLC44A1 cDNA (Sino Biologics, # RG80408-CM), pCMV-SPORT6-SLC44A2 cDNA (Genomics Online, # ABIN3822596), or empty vector (pcDNA4His-Max B, # V86420) using Lipofectamine 2000 (Invitrogen). Transfections with 30 nM SLC44A2 siRNA (Santa Cruz Biotechnology, # sc-62163) was with siPORT lipid transfection agent. Transfection with 80 pmol of OCT1/SLC22A1 (Santa Cruz Biotechnology, # sc-42552) was done with siRNA Transfection Reagent (Santa Cruz Biotechnology, # sc-29528) and Transfection Medium (Santa Cruz Biotechnology, # sc-36868) as per manufacture’s protocol. DNA Ladder used was O'GeneRuler Express (Thermo Scientific).

### Immunoblotting

Cells were washed 3× with PBS and subjected to a lysis buffer (25 mM Tris, 15% glycerol, 1% Triton X-100, 8 mM MgCl_2_, 1 mM DTT, protease inhibitor cocktail and phosphatase inhibitor cocktail) at 4 °C for 30 min. Protein concentration was determined with the bicinchoninic assay (Pierce). The LV58 (N terminus) and ENS-627 (C terminus) antibodies (both 1:500 in 5% skim milk in TBS-T) were used to detect the 72 kDa CTL1 protein under native (nondenaturing) conditions. Samples were mixed with loading buffer (62 mM Tris-HCl, 0.01% bromophenol blue, and 10% glycerol) and separated by PAGE at 120 V for 1.5 h. CTL1, CTL2 (Abnova; 1:200 in 5% skim milk in TBS-T), and β-tubulin (Cell signaling; 1:1000 in 5% skim milk in TBS-T) were resolved on an 8% native gel, and proteins were transferred onto polyvinylidene difluoride membranes (Pall Canada) by a semidry transfer system and stained with Ponceau S. Membranes were blocked in 5% skim milk in tris-buffered saline-Tween 20 (TBS-T) solution and then incubated with primary antibodies (1:500 in 5% skim milk in TBS-T) overnight at 4 °C. Membranes were washed with TBS-T and then incubated with an anti-rabbit horseradish peroxidase–conjugated secondary antibody (New England Biolabs, 1:10,000 in 5% skim milk in TBS-T) for 2 h. Membranes were washed in TBS-T, and proteins were visualized using a chemiluminescent substrate (Sigma-Aldrich). Precision Plus Dual Color (BioRad) was used as the immunoblotting size marker.

### Mitochondrial isolation

Mitochondria were isolated as initially described (5). In brief, cells were incubated for 20 min in ice-cold RSB swelling buffer and homogenized; 19 ml MS buffer was added, and the cell homogenate was centrifuged at 2500 rpm for 5 min. This step was repeated twice, and the final supernatant was centrifuged at 12,500 rpm. The resulting pellet was resuspended in MS buffer. The mitochondrial purity was determined using β-tubulin as a whole cell control and a mitochondria-specific biomarker cytochrome c oxidase subunit IV (COXIV), the last enzyme in the mitochondrial respiratory chain (45).

### Statistical analysis

All measurements are expressed as means from quadruplets ± SD. Statistical analysis was performed using GraphPad Prism software (GraphPad, Inc). Data were subjected to Students *t* test. Differences were considered statistically significant at *p* < 0.05.

## Data availability

All study data are included in the article.

## Conflict of interest

The authors declare that they have no conflicts of interest with the contents of this article.
